# Characterization of Crystals in Ciliate *Paramecium bursaria* Harboring Endosymbiotic *Chlorella variabilis*

**DOI:** 10.1007/s00284-024-03793-8

**Published:** 2024-07-13

**Authors:** Yuuki Kodama, Ayasa Kitatani, Yuriko Morita

**Affiliations:** 1 Institute of Agricultural and Life Sciences, Academic Assembly, Shimane University, Matsue-shi, Japan; 2 Department of Life Sciences, Faculty of Life and Environmental Sciences, Shimane University, Matsue-shi, Japan

## Abstract

Protists, including ciliates retain crystals in their cytoplasm. However, their functions and properties remain unclear. To comparatively analyze the crystals of *Paramecium bursaria*, a ciliate, associated with and without the endosymbiotic *Chlorella variabilis*, we investigated the isolated crystals using a light microscope and analyzed their length and solubility. A negligible number of crystals was found in *P. bursaria* cells harboring symbiotic algae. The average crystal length in alga-free and algae-reduced cells was about 6.8 μm and 14.4 μm, respectively. The crystals of alga-free cells were spherical, whereas those of algae-reduced cells were angular in shape. The crystals of alga-free cells immediately dissolved in acids and bases, but not in water or organic solvents, and were stable at – 20 °C for more than 3 weeks. This study, for the first time, reveals that the characteristics of crystals present in the cytoplasm of *P. bursaria* vary greatly depending on the amount of symbiotic algae.

## Introduction

Many unicellular protists, such as ciliates and rhizopods, possess crystals in their cytoplasm [1]. Crystals have been easily observable under a polarizing microscope for over a century. Previous studies have revealed the characteristics of the crystals in some protists [1–3]. A previous study revealed that crystals in the freshwater ciliate *Paramecium tetraurelia* contain calcium struvite [4]. The freshwater ciliate *Spirostomum ambiguum* maintains calcium phosphate in its cytoplasm [5], and the marine dinoflagellate *Gonyaulax polyedra* and marine ciliate *Parauronema actum* retain purines, such as guanine and hypoxanthine [6, 7]. Differences in feeding conditions (i.e., in axenic medium or in cultures fed on bacteria) may have contributed to the differences in crystal composition 3. These studies suggest that the habitat or environment affects the crystal components retained in protists. Furthermore, a recent report led to significant advances in the study of crystals [8]. Using Raman microscopy, Pilátová et al. [8] detected cellular crystalline inclusions composed mainly of purines in 77% of more than 200 species from all the major eukaryotic supergroups. Anhydrous guanine crystals have also been found in *Paramecium* sp. [8]. There are three hypotheses concerning the role of ciliate crystals: firstly, they serve as a pathway for excretion of excess purines [7]; secondly, they act as a storage reservoir for purines and organic nitrogen, which can be utilized during periods of starvation [3]; thirdly, they sequester nitrogenous waste, preventing its excretion into the environment, where it could act as a chemotactic signal to predators [3]; however, the chemical and physiological nature of the crystals has not been elucidated [9].

It is known that *Paramecium bursaria* forms endosymbiotic relationships with *Chlorella vulgaris, Chlorella variabilis, Chlorella sorokiniana* and *Micractinium conductrix* [10]. Ciliates and algae exhibit mutualistic associations. The host cell provides algae with nitrogen and CO_2_ [11–13], and the algae are protected from infection by the *Chlorella* virus within the host [14], whereas the algae provide the host with photosynthetic products, mainly maltose and oxygen [15, 16]. Both *P. bursaria* and symbiotic *Chlorella* spp. hold the ability to live unpartnered. Endosymbiosis can be re-established between alga-free *P. bursaria* cells and symbiotic *Chlorella* sp. cells isolated from algae-bearing host cells [17, 18]. The genomes of the symbiotic *C. variabilis* [19] and *P. bursaria* [20] were sequenced, and both protists are now considered as models for the study of endosymbiosis [21]. Similar to other protists, *P. bursaria* retains crystals in the cytoplasm; however, as mentioned above, many symbiotic algae are also present in the same place*.* Hence, the following questions were raised: Can crystals and symbiotic algae coexist in their cytoplasm? Do symbiotic algae affect crystal abundance or length?

To comparatively analyze the intracellular crystals between *P. bursaria* cells with and without symbiotic algae, crystals were isolated from alga-free and algae-reduced *P. bursaria* cells, that is, cells in which the symbiotic algal number was artificially reduced by culturing under constant dark conditions. Subsequently, the length and shape of the crystal structures were then analyzed, and as was the solubility of the crystals in various solutions, similar to that examined for crystals of *Paramecium multimicronucleatum* [1].

## Materials and Methods

### *Paramecium* and Algal Strains and Cultivation

Two *P. bursaria* strains were used in this study: alga-free Yad1w and algae-bearing Yad1g1N (syngen R3 [10, 22, 23], mating type I). Strain Yad1g1N was produced by infecting Yad1w cells with the cloned symbiotic *Chlorella* sp. 1 N cells [24]. Strain 1N was identified as *C. variabilis* by rbcL gene analysis [25]. Moreover, *P. multimicronucleatum* strain YM-25 (syngen 2, mating type III or IV [cycler]) was used. All *Paramecium* strains were cultured using a sterilized 30 ml test-tube in red pea (*Pisum sativum*) extract culture medium [26] in Dryl’s solution ([27], KH_2_PO_4_was used instead of NaH_2_PO_4_·2H_2_O), which was inoculated with non-pathogenic *Klebsiella aerogenes* (ATCC35028) 1 d before use [28]. For ordinary cultures, several hundred cells of all *Paramecium* species were inoculated into 2 mL aliquots of the culture medium in test-tubes. Subsequently, 2 mL aliquots of fresh culture medium were added every day for 12 days. Cultures in the early stationary growth phase were used in the experiments 1 day after the final feeding and were cultivated at 25 ± 1 °C. The cultures at this time were no longer turbid, which means that most of the bacteria had been eaten. In addition, few digestive vacuoles were observed in the cells. Algae-bearing cells were cultured under fluorescent lighting maintained at 20–30 μmol photons m^−2^s^−1^ using an incandescent lamp under 24 h constant light (LL). To reduce the number of symbiotic algae, algae-bearing cells were incubated with food bacteria under constant dark conditions for 18–30 days. All *Paramecium* strains used in this study were provided by the NBRP *Paramecium* Laboratory, Yamaguchi University, with support, in part, by the NBRP of the Ministry of Education, Culture, Sports, Science, and Technology (MEXT) (http://nbrpcms.nig.ac.jp/paramecium/?lang=en ).

### Microscopy

*Paramecium* cells and intracellular crystals were visualized using a differential interference contrast (DIC) microscope (BX53; EVIDENT, Tokyo, Japan), images were captured using an Olympus DP74 digital camera (EVIDENT, Tokyo, Japan), and crystal luminance was analyzed using Olympus cellSens Dimension software (EVIDENT, Tokyo, Japan). For each strain, 10–12 *Paramecium* cells were analyzed. The crystal length was measured using the ImageJ software (National Institute of Health, Bethesda, MD, USA). The crystal morphologies were determined manually by observing the photomicrographs of each crystal. An inverted phase-contrast microscope (CK2; Olympus, Tokyo, Japan) was used to observe the crystals. Images were obtained using an i-NTER LENS device (Micronet Inc., Japan).

### Isolation of the Crystals from *P. bursaria* Cells

Crystals were isolated using a previously described method [25]. Approximately 300 ml of both alga-free and algae-reduced *P. bursaria* cells were strained through two layers of Kimwipes to remove large debris. The cells were then transferred to a plastic beaker through a nylon mesh with a pore size of 15 μm. The cell pellet was washed with 100 mL phosphate-buffered saline (PBS) (137 mM NaCl, 2.68 mM KCl, 8.1 mM NaHPO_4_·12H_2_O, 1.47 mM KH_2_PO_4_, pH 7.2), and paramecia were harvested in a plastic beaker. Alga-free or algae-reduced *P. bursaria* cells (5 × 10^4^ cells) were lysed using the EzRIPA Lysis kit (ATTO, Tokyo, Japan) according to the manufacturer’s protocol. Numerous intracellular crystals were detected in the insoluble precipitates of alga-free and algae-reduced *P. bursaria*. The algae-bearing *P. bursaria* cells in the insoluble precipitates showed many symbiotic algae.

### Solubility of the Crystals

The solubility of the crystals in 11 different reagents was analyzed according to the method reported by [1]. The isolated crystals were placed on glass slides and air-dried. 20–30 μL of each reagent was placed between the cover glass and slide glass, and its effect on the crystals was observed using microscopy. To explore the solubility of the crystals at different temperatures, we suspended 200 μL of crystals isolated from alga-free *P. bursaria* in Dryl’s solution [27] in 1.5 mL tubes, and the samples were incubated at 25, 22, 16, 10, 4, and − 20 °C for 24 h, 48 h, 72 h, 1 week, and 3 weeks. Subsequently, the crystals were obtained, observed, and analyzed as previously described above. All experiments were conducted at 25 ± 1 °C.

### Statistical analysis

Mann–Whitney *U* test and two-tailed Fisher’s exact test were used to analyze the data. Statistical significance was considered at *P* < 0.001 [indicated by asterisks (∗∗∗)]. All data are expressed as mean (± SD). All statistical analyses were conducted using R software (R Ver 4.1.3) [29].

## Results

### The Abundance of the Crystals in the Cytoplasm of *P. bursaria *with and without Symbiotic Algae and *P. multimicronucleatum*

**Fig. 1:**
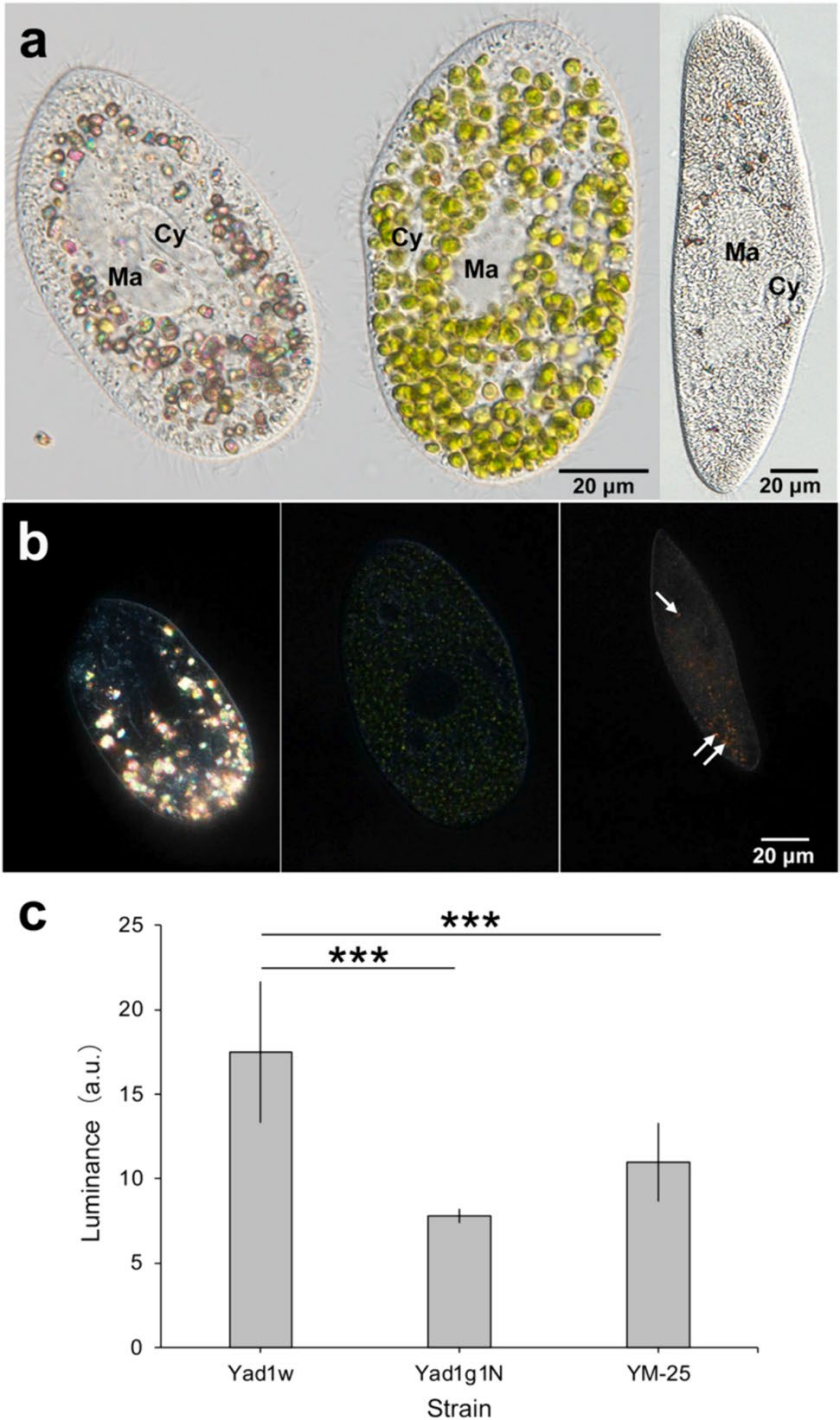
DIC micrographs showing alga-free Yad1w (**a**, left), algae-bearing Yad1g1N (**a**, middle) *Paramecium bursaria* cells, and *P. multimicronucleatum* strain YM-25 cell (**a**, right). Many intracellular green symbiotic algae were visible in algae-bearing *P. bursaria* cell. Many crystals were observed in alga-free cell, but few crystals were observed in algae-bearing cell. A few small crystals have been observed in *P. multimicronucleatum*. Cy, cytopharynx; Ma, macronucleus. DIC photomicrographs adjusted to highlight crystals (**b**). The brightness of all images was increased because the small and dark crystals of alga-bearing *P. bursaria* (**b**, middle) and *P. multimicronucleatum* (**b**, right) were difficult to observe. Numerous glowing crystals were observed in Yad1w, especially at the posterior end (**b**, left). In Yad1g1N, fairly small crystals were observed (**b**, middle). A few crystals were also present in YM-25 (**b**, right, white arrows); however, they were less bright) Comparative mean luminosity of intracellular crystals in three *Paramecium* strains (**c**). The mean luminosity and standard deviation of the crystals of each strain are presented, and the mean luminosity of Yad1w was the highest, followed by those of YM-25 and Yad1g1N. Significant differences in the mean luminosity of Yad1w and Yad1g1N and Yad1w and YM-25 were detected (Mann–Whitney *U* test, ***P < 0.001). This result was in good agreement with the microscopic observations in (**a**, **b**). For each strain, ten to twelve *Paramecium* cells were analyzed

Figure 1 shows a typical DIC image of alga-free (Fig. 1a, left) and algae-bearing (Fig. 1a, middle) *P. bursaria* and *P. multimicronucleatum* strain YM-25 (Fig. 1a, right). *Paramecium multimicronucleatum* is easy to observe because it is a large species in the genus *Paramecium*. Because the solubility of its intracellular crystals was examined in a previous study [1], we compared the abundance of intracellular crystals in *P. bursaria* with that in *P. multimicronucleatum.* Several green symbiotic *C. variabilis* cells were observed in algae-bearing Yad1g1N cells (Fig. 1a, middle). Many crystals (polarized orange granules) were identified in alga-free Yad1w cells (Fig. 1b, left). The crystals were localized at the posterior end of the cell rather than beneath the cell cortex, and most crystals moved inside the cell via *P. bursaria* cytoplasmic streaming, as demonstrated in our previous study [30]. In contrast, a negligible number of crystals were found in algae-bearing cells (Fig. 1b, middle). *P. multimicronucleatum* (Fig. 1b, right) cells exhibited a few small crystals.

Luminance analysis using photomicrographs revealed that the average luminance of intracellular crystals was 17.5, 7.8, and 12.0 in Yad1w, Yad1g1N, and *P. multimicronucleatum* strain YM-25 cells, respectively (Fig. 1c). Moreover, as shown in Fig. 1b (left), the red color after adjusting the objective Nomarski prism is visible on the crystals in the Yad1w cells, indicating high luminosity.

### The Crystals in Algae-Reduced *P. bursaria*

**Fig. 2:**
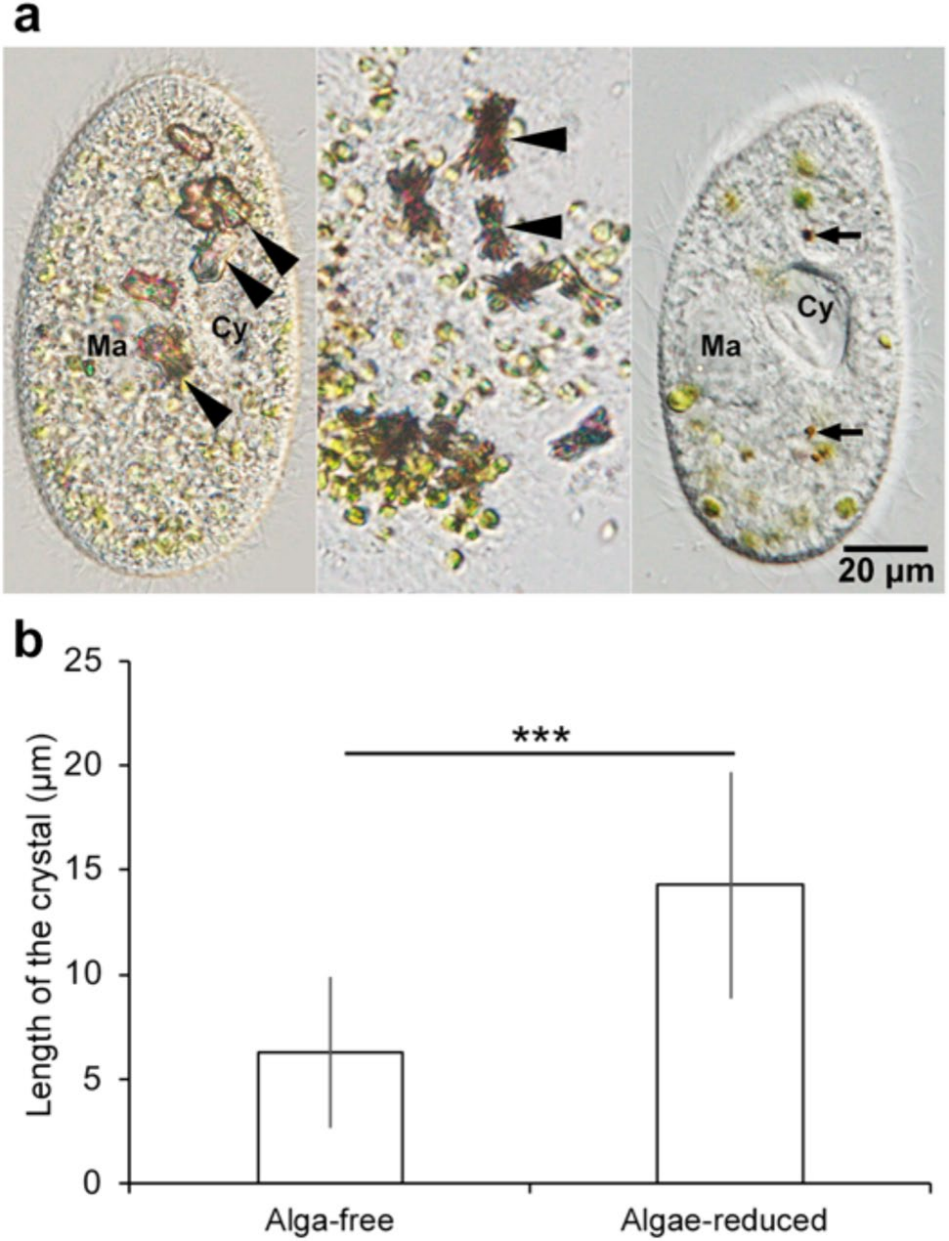
DIC micrographs of algae-reduced *P. bursaria* cells cultivated under constant dark conditions for 18 days with feeding (**a**, left). Crystals obtained from disrupted cells (**a**, middle panel). Algae-reduced *P. bursaria* cells were cultivated under constant dark conditions for 18 days without feeding (**a**, right). Black arrowheads (**a**, left and middle) show larger crystals than alga-free *P. bursaria* cells (Fig. 1a, left). No crystals were observed without feeding; however, some digested brown algae coexisted in the cell (**a**, right, black arrow). Cy, cytopharynx; Ma, macronucleus. **b** Length of the crystal of alga-free and algae-reduced *P. bursaria* cells; a total of 660 and 117 crystals of alga-free and algae-reduced cells were measured. The average length of the crystals of algae-reduced *P. bursaria* was more than twice that of the alga-free cells. Error bars indicate standard deviation (SD). Asterisks indicate significant differences (two-sided Fisher’s exact test, ***P < 0.001)

The cultivation of algae-bearing *P. bursaria* under constant darkness can induce algal reduction [31]. After 18–30 days of cultivation, the number of symbiotic algae decreased drastically (Fig. 2a, left). Most of the observed cells retained 10 or fewer algae per cell. Interestingly, accompanied by algal reduction, intracellular crystals appeared (Fig. 2a, left), which were conspicuously larger than those found in alga-free cells (Fig. 2a, middle). Similar results were obtained from the images available in [32]. Furthermore, crystals did not appear when the algae-bearing cells were incubated under constant dark conditions without food bacteria (Fig. 2a, right). This result is in agreement with that of Wichterman [33], who demonstrated the complete disappearance of crystals within 1 or 2 days when the cells were maintained under starvation. Cultivation of alga-free *P. bursaria* cells under the dark condition did not affect to their crystals (data not shown). This means that the dark treatment alone did not induce an increase in the number of crystals. While analyzing the length of the crystals isolated from alga-free cells using ImageJ (Fig. 2b, left bar), an average length of 660 crystals was detected to be 6.3 μm (SD = 3.6, *n* = 4; ranging from 1.0 to 27.2 μm). A total of 117 crystals isolated from algae-reduced cells (Fig. 2b, right bar) exhibited an average length of 14.3 μm (SD = 5.4, *n* = 3; ranging from 3.4 μm to 33.5 μm), which was more than 2 times longer than that recorded in alga-free cells. As reported by [1], the crystals of *P. multimicronucleatum*, the larger species in the genus *Paramecium*, are 0.2–25 μm in length, which included significantly smaller crystals than that of algae-reduced *P. bursaria* cell.

#### Cellular Content of Alga-Free and Algae-Bearing *P. bursaria* in Insoluble Precipitate

**Fig. 3:**
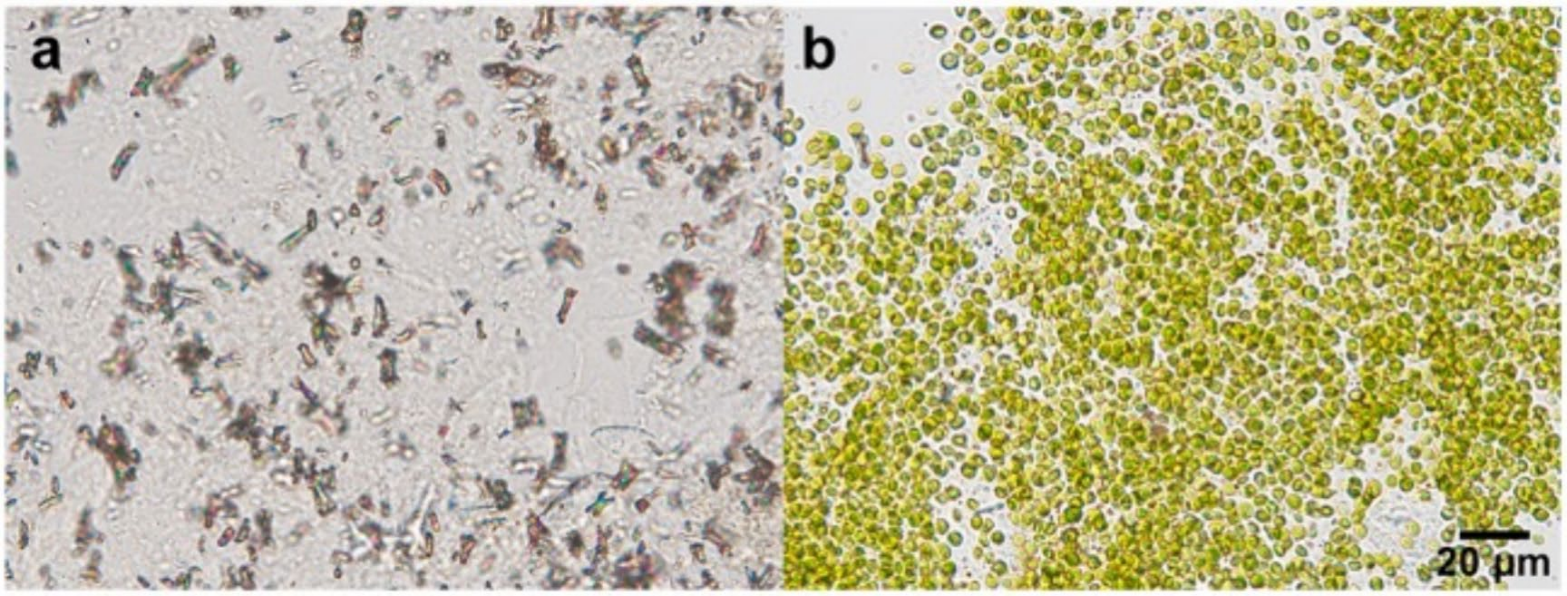
Isolated crystals derived from alga-free *P. bursaria* cells (**a**). Many crystals were visible in the precipitate of the protein extraction with RIPA Lysis buffer (described in the Materials and Methods section). No crystals were visible in the same fraction of the algae-bearing *P. bursaria* cell lysate and many symbiotic algae can be obtained instead (**b**)

Figure 3 shows alga-free and algae-bearing *P. bursaria* cellular contents in the insoluble precipitates obtained after cell lysis. Many intracellular crystals were detected in the alga-free cell lysate (Fig. 3a) but were absent in the algae-bearing cell lysate (Fig. 3b). Numerous symbiotic algae were observed in algae-bearing cell lysates (Fig. 3b).

### Morphology of the Crystals Isolated from Alga-Free and Algae-Reduced *P. bursaria*

**Fig. 4:**
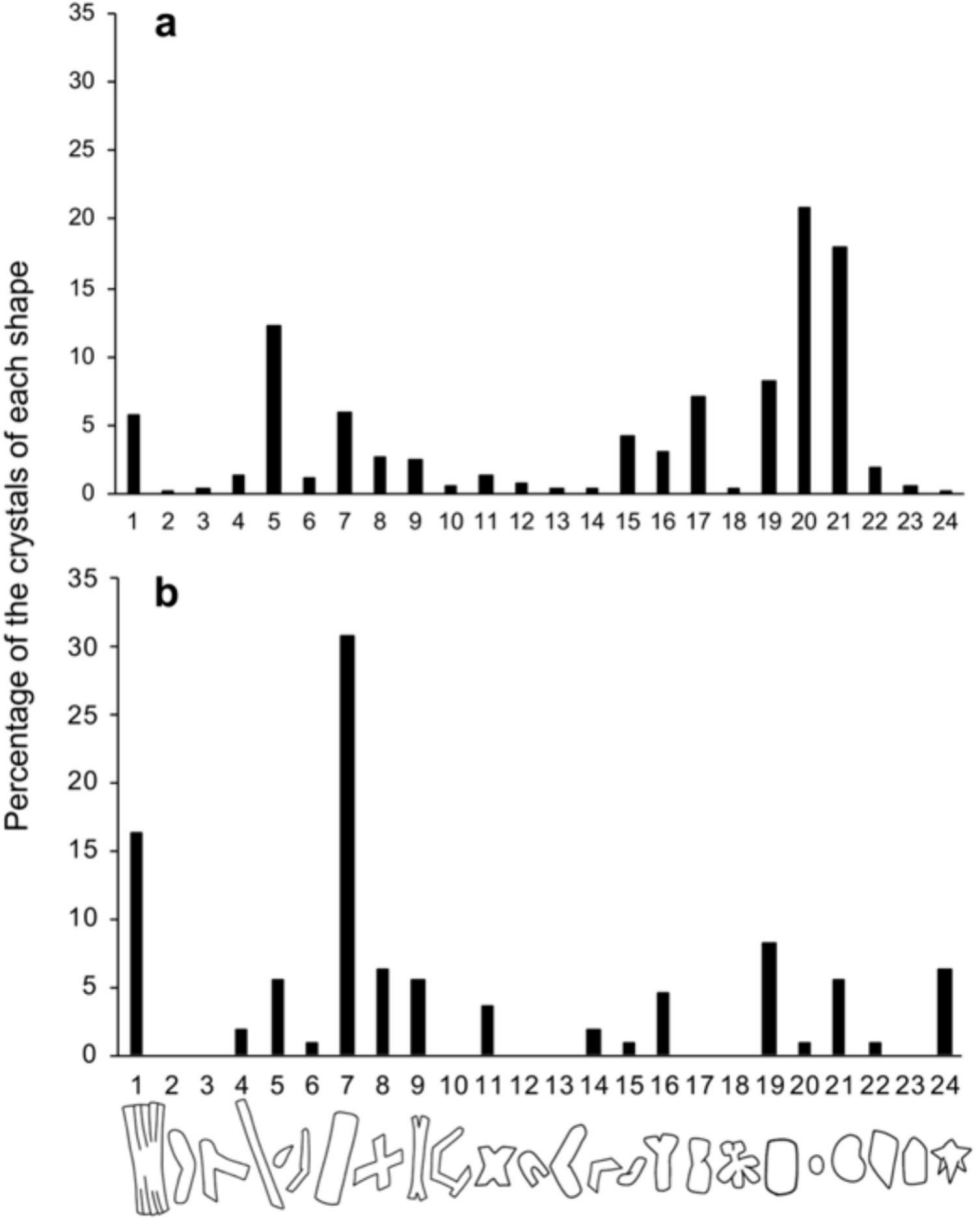
Morphology of crystals isolated from alga-free (**a**) and algae-reduced (**b**) *P. bursaria*. The numbers on the x-axis refer to the crystal shape type. The morphologies of 660 crystals of alga-free cells and 117 crystals of algae-reduced cells were observed. Note that the morphology of the crystal was changed by the algal reduction due to algal digestion

The crystals isolated from alga-free and algae-reduced *P. bursaria* were classified into 24 types (x-axis in Fig. 4), based on their morphology. Among the analyzed 660 crystals derived from alga-free *P. bursaria*, many rounded crystals, such as types 20 and 21, were observed (Fig. 4a); moreover, several drop-shaped (type 5) and plate-shaped crystals (type 1) were identified.

Among the 117 crystals isolated from algae-reduced *P. bursaria* (Fig. 4b), many angular-shaped crystals, such as rod-shaped (type 7) and plate-shaped (type 1) crystals, were detected, whereas crystals categorized as types 2, 3, 10, 12, 13, 17, 18, and 23 were not observed (Fig. 4b).

### Solubility of the Crystals Isolated from Alga-Free *P. bursaria*

**Fig. 5:**
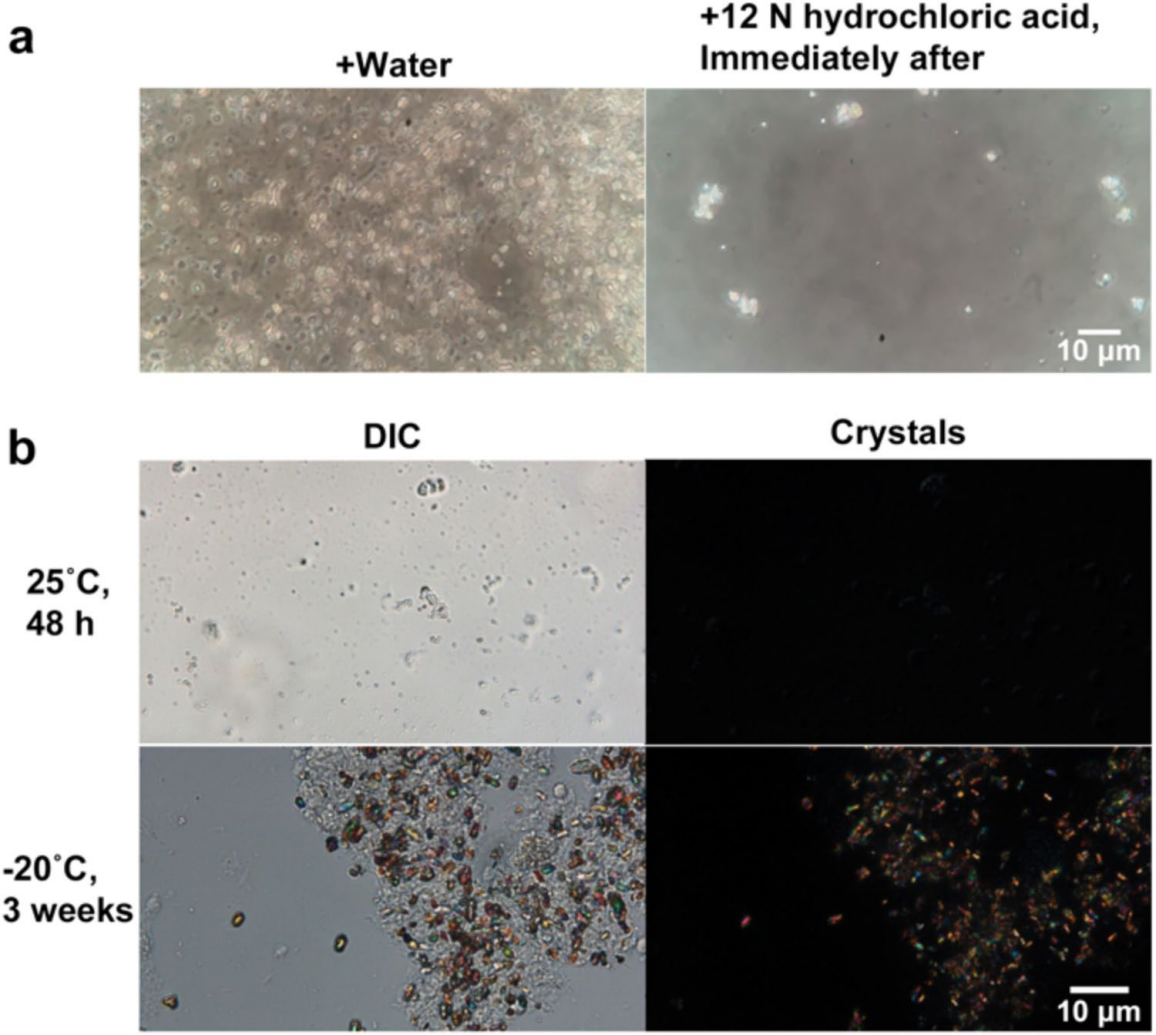
**a** Solubility of crystals isolated from alga-free cells using various reagents (**a**, left) shows a typical image of crystals that were not dissolved after adding the reagent. (**a**, right) shows a typical image of the crystal sample instantly dissolved after the addition of 12 N hydrochloric acid. **b** Photomicrographs showing crystals stored at different temperatures (**b**). DIC microscopy image (**b**, upper, left) of crystals stored at 25 °C recorded after 48 h; photomicrograph of crystals (**b**, upper, right). These crystals were non-reflective and completely dissolved. DIC microscopy image (**b**, lower, left) of crystals recorded 3 weeks after storing at − 20 °C; micrograph of crystals (**b**, lower, right). The crystals were well-preserved

Table 1 summarizes the solubilities of the crystals isolated from alga-free cells using various reagents. The crystals were lysed immediately in 12 N hydrochloric acid and 8 N sodium hydroxide; however, in water, lysis was not observed within 15 min, whereas hot water (80 °C) slowly induced lysis. Figure 5a (left) shows that the crystals maintained their shapes in reagents that were insoluble. In contrast, the crystals dissolved immediately after dropping into the effective reagents (Fig. 5a, right). Furthermore, the remaining crystals, some of which are visible in the marginal area of Fig. 5a (right), were completely dissolved in the reagents by gentle pipetting.

**Table 1:** Solubility of the crystals of alga-free *Paramecium bursaria*. The solubility was determined according to Berngeimer (1938)

Water	Hot water (80 °C)	Glycerol	Ether	95% Ethanol	0.1N HCl	12N HCl	Acetic acid	NH_4_OH	0.1N NaOH	8N NaOH
×	▲	×	×	×	×	○	×	△	×	○

○ crystals dissolve immediately

△ dissolve within 2 min

▲ dissolve within 15 min

× crystals do not dissolve in 15 min

Table 2 summarizes the solubilities of alga-free *P. bursaria* crystals at different temperatures. The crystals dissolved after 48 h at 25 °C are depicted in Fig. 5b (upper); however, the time required for dissolution increased with decreasing storage temperature, and the crystals stored at − 20 °C retained their shape over 3 weeks (Fig. 5b, lower). The luminescence of the crystals changed immediately before melting and the crystals appeared white. Similar results were obtained when the crystals were stored without Dryl’s solution (data not shown).

**Table 2:** Solubility of the crystals of alga-free *Paramecium bursaria* at different temperatures

	24 h	48 h	72 h	1 week	3 weeks
25 °C	++	−	−	−	−
22 °C	++++	+	+	−	−
16 °C	+++++	+++++	+++++	+	−
10 °C	+++++	+++++	+++++	++	−
4 °C	+++++	+++++	+++++	++++	+
− 20 °C	++++	++++	++++	++++	++++

The crystals immediately after isolation were labeled + + + + +

As the lysis progresses, ++++ , ++ , +++ , ++ , and when no crystals are observed at all, it is designated as -

## Discussion

In this study, we demonstrated that maintenance of intracellular algal symbiosis decreased crystal retention in the host cytoplasm. Although the mechanism underlying the reduction in the abundance of intracellular crystals is unclear, two potential causes associated with the infection process of *Chlorella* sp. in alga-free *P. bursaria* cells have been proposed: first, the host cells may excrete crystals into the culture solution; second, the presence of guanosine metabolites (i.e., crystal precursors), which is a prerequisite for the establishment of endosymbiosis. We observed crystals frequently excreted through the host cytoproct during algal endosymbiosis in alga-free *P. bursaria* (Kodama, unpublished data), which supports the first possibility; however, the exocytosis of intact crystals has never been reported in *Paramecium* [9]. Since the crystals in the ciliates are “stores’ of waste products of guanosine metabolism abundant under rich food supply and scarce under starvation, the low number of crystals in algae-bearing *P. bursaria* may be explained by the lack of excess nutrients due to their symbiotic *Chlorella* sp. Long crystals formed in the case of cultures kept in the dark may be explained by the appearance of excess nutrients in the ciliate because of autophagy of the algae in the dark. In the course of autophagy the amount of metabolites must increase. The absence of crystal formation in cultures kept in the dark without feeding was in good agreement with this explanation. Thus, the presence of crystals or guanosine metabolites (i.e., crystal precursors), rather than the presence of crystals, may be a prerequisite for establishing a symbiotic relationship. We performed an infection experiment with the expectation that addition of crystals isolated from alga-free *P. bursaria* to *Chlorella* sp. would increase the rate of algal endosymbiosis. However, we found no difference in endosymbiosis rates between alga-free *P. bursaria* ingesting a mixture of *Chlorella* sp. and crystals and control *P. bursaria* cells ingesting only *Chlorella* sp. (Kitatani and Kodama, unpublished data). A previous study demonstrated that the number of host mitochondria and trichocysts was significantly reduced with increasing numbers of endosymbiotic algae [24, 25, 34]. Similarly, algal reinfection may decrease the number of host crystals. The entry of algae into host *P. bursaria* cells may trigger the ejection of crystals occupying a major space in the cytoplasm to secure the space required for endosymbiotic association. Recently, [30] revealed a significantly decreased abundance of crystals, which almost disappeared when the original symbiotic *Chlorella* sp. was used to inoculate alga-free *P. bursaria* cells, whereas free-living *Chlorella* sp. induced a smaller decrease in the number of crystals, thus supporting the second possibility.

Pilátová et al. [8] suggested “that purine crystals, possibly present in the last eukaryotic common ancestor, were the first type of biocrystals in eukaryotes contingent on the emergence of cell compartmentalization in early eukaryotes. Owing to the low-solubility and high-capacity, purine inclusions possibly have emerged through an adaptation to nitrogen detoxification, protection against exposure to high levels of ammonia or nitrates, and utilization of vacuoles as a versatile sequestration space.”Although the role of crystals in the process of establishing or maintaining endosymbiosis between *P. bursaria* and zoochlorellae remains unclear, this is the first study to report the effect of endosymbiotic algae in *P. bursaria* on the prevalence of host cytoplasmic crystals. Daniels [35] demonstrated that starving for 1–2 days induces complete disappearance of crystals from the *Amoeba* and crystals reappear when the *Amoeba* are fed with prey. Therefore, it has been speculated that crystals act as a source of nutrients for *Amoeba* [35]. In this study, algae-bearing *P. bursaria* Yad1g1N contained few crystals (Fig. 1a, middle); however, the luminosity of Yad1g1N was also 7.8 (Fig. 1c), which may be attributed to the crystals in *Chlorella*, as demonstrated by [8]. A photosynthetic product, mainly maltose, is provided to the host *P. bursaria* [15, 36]; hence, starvation is not expected in host cells under constant light conditions. In fact, algae-bearing *P. bursaria* grow faster than alga-free cells under starvation conditions [31]. The absence of crystals in the cytoplasm of algae-bearing cells, even when nutrients are available, suggests that crystalline components may be used to maintain endosymbiosis; however, more research is needed in the future.

Mycosporine-like amino acids (MAAs) produced by symbionts may be related to host protection through the accumulation of sunscreen compounds in tissues [37]. The existence of MAAs in symbiotic ciliates has been reported in marine and freshwater species; however, the presence of MAA has not been confirmed in algae-bearing *P. bursaria* [37]. Summerer et al. [38] reported that exposure to artificial UV radiation (UVR) + photosynthetically active radiation (PAR) and ‘‘high’’ PAR (160 mmol m^−1^ s^−1^) showed an immediate aggregation of algae-bearing *P. bursaria* into several dense ‘‘spots’’ of approximately 1–3 mm in diameter in a Petri dish. Furthermore, Summerer et al. [38] reported that *P. bursaria* can protect against UV damage by accumulation as well as by symbiont dislocation. One of the functions of protist crystals is to protect them from UV radiation [39]. As an alternative to UV protection by symbiotic zoochlorellae, alga-free *P. bursaria* may increase its retained crystals.

Although it has also been reported that alga-free *P. bursaria* is found in nature [41], some algae were present in *P. bursaria* cells collected from the field (data not shown). Therefore, it can be said that the presence of endosymbiotic zoochlorellae is typical for *P. bursaria*. When the number of algae is artificially reduced by culturing them under constant dark conditions with food bacteria, crystals may be generated from substances obtained during the digestion processes of both algae and bacteria, and they may be stored in the cytoplasm. Thus, storing the crystals in the cytoplasm of alga-free *P. bursaria* cells may provide opportunities for endosymbiosis.

Why do crystals in the cytoplasm became larger as the number of symbiotic algae decreases (Fig. 2)? Analysis of the crystal length revealed that the minimum length was 0.2 μm, while the maximum length was 25 μm in *P. multimicronucleatum*, the larger species in the genus *Paramecium* [1]. As shown in Fig. 2b, in the crystals isolated from the algae-reduced cells, the minimum length was 3.4 μm and the maximum length was 33.5 μm; the maximum length was larger than that of *P. multimicronucleatum* (0.2–25 μm; [1]). The crystal structure is surrounded by a membrane [9]; hence, the crystals are considered to potentially grow inside the vesicle and increase due to the binding of crystals wrapped in another vesicle membrane. It is possible that the components obtained from the digestion of symbiotic algae by *P. bursaria* are involved in this growth method. Figure 2a (left and middle) shows crystals grown after algal digestion under constant dark conditions. Crystals were absent in the absence of bacteria (Fig. 2a, right). This interesting change accompanied by algal reduction possibly indicates that the crystals are not made from algal-digested components alone, but involve bacterial-digested components. While analyzing the size of the crystals, Hausmann et al. [9] reported that *Paramecia* fed on bacteria contained small crystal particles; after being fed on protein or meat extracts, they contained numerous large crystals. Foraminifers contained crystals after feeding on copepods or ciliates; however, no crystals were found after a diet restricted to diatoms. However, the direct relationship between food digestion and crystal formation has yet to be determined.

Regarding morphology of the crystals, the crystals isolated from the alga-free *P. bursaria* had a round shape (Fig. 4a), whereas those isolated from the algae-reduced *P. bursaria* had angular shapes, such as rods and plates (Fig. 4b). The crystals of protists are one of the criteria used for species identification [40]; however, our results showed that the size and shape of the crystals in *P. bursaria* changed significantly with changes in the number of symbiotic algae (Fig. 4). Interestingly, alga-free *P. bursaria* cells fed compatible *Chlorella* sp., such as the original symbiotic algae, lost their intracellular crystals during the algal infection process, but not when fed less compatible *Chlorella* sp., such as free-living *Chlorella* sp. [30]. Since one of the roles of crystals is as a storage reservoir for purines and organic nitrogen [3], the amount and composition of photosynthetic products of symbiotic algae may affect the host crystals.

Crystals of seven protist species (*Mayorella* sp., *Cochliopodium bilimbosum, Trichamoeba villosa, Chaos diffluens, Chilomonas paramecium, Halteria grandinella*, and *Paramecium multimicronulceatum*) have been shown to dissolve immediately after treatment with strong acids or bases [1]. Furthermore, their crystals were dissolved in water within 15 min. As shown in Table 1, *P. bursaria* crystals also showed a similar trend of high solubility in strong acids and bases, but they were not dissolved in water. A comparative study of the solubilities and melting points of paramecium and other protist crystals in various solutions may help predict the constituents or functions of the crystals.

As shown in Fig. 3a, we successfully isolated a large number of high-purity crystals from the *Paramecium* cells, revealing high stability at − 20 °C (Table 2). The crystals contain guanine, suggesting that successful large-scale cultures of *P. bursaria* could lead to an environmentally safe fertilizer.

## Conclusion

To our knowledge, this is the first report to reveal an interesting association between an endosymbiotic alga and crystals found in *P. bursaria* cells, helping to understand the important functional properties of the crystals. We propose two possible reasons for the presence of more crystals in alga-free *P. bursaria* than in algae-bearing cells: (1) Crystalline components may have been utilized to maintain endosymbiotic algae, as indicated by the length of the crystals of algae-reduced *P. bursaria*, which increased up to twice the length of alga-free cells. (2) The crystals may be excreted from host cells during the algal reinfection process. Furthermore, a large number of high-purity crystals were successfully isolated from *Paramecium* cells. Moreover, the crystals of *P. bursaria* were soluble in strong acids and bases and were suitable for long-term storage at − 20 °C though ciliates cannot survive freezing. Future studies comparing the solubility, melting point, and components of *Paramecium* in various solutions may help to predict the constituents or functions of the crystals.

## Data Availability

The datasets used in this study are available from the corresponding author upon reasonable request.
